# Subjective reward processing and catechol-*O*- methyltransferase Val158Met polymorphism as potential research domain criteria in addiction: A pilot study

**DOI:** 10.3389/fpsyt.2022.992657

**Published:** 2022-10-14

**Authors:** Nico Rohlfing, Udo Bonnet, Indira Tendolkar, Anke Hinney, Norbert Scherbaum

**Affiliations:** ^1^Department of Addictive Behaviour and Addiction Medicine, LVR-Hospital Essen, Hospital of the University of Duisburg-Essen, Essen, Germany; ^2^Department of Psychiatry and Psychotherapy, LVR-Hospital Essen, Hospital of the University of Duisburg-Essen, Essen, Germany; ^3^Department of Psychiatry, Psychotherapy and Psychosomatic Medicine, Evangelisches Krankenhaus Castrop-Rauxel, Academic Teaching Hospital of the University of Duisburg-Essen, Essen, Germany; ^4^Donders Institute for Brain, Cognition and Behaviour, Centre for Medical Neuroscience, Radboud University Nijmegen, Nijmegen, Netherlands; ^5^Department of Child and Adolescent Psychiatry, Psychosomatics and Psychotherapy, University Hospital Essen, University of Duisburg-Essen, Essen, Germany

**Keywords:** subjective reward processing, COMT Val158Met polymorphism, research domain criteria, insula, gain expectancy

## Abstract

The Research Domain Criteria (RDoC) approach seeks to understand mental functioning in continuous valid dimensions ranging from functional to pathological. Reward processing is a transdiagnostic functioning domain of the RDoC. Due to prototypical abnormalities, addictions are especially applicable for the investigation of reward processing. Subjective reward processing is challenging to determine and differs between genotypes of the catechol-*O*-methyltransferase gene (COMT) Val158Met polymorphism for incomparable daily life experiences. Thus, we implemented the monetary incentive delay (MID) task with comparable reward cues and visual analog scales (VAS) to assess subjective reward processing in male abstinent cannabis-dependent individuals (*N* = 13) and a control group of nicotine smokers (*N* = 13). COMT Val158Met genotypes were nominally associated with differences in cigarettes smoked per day and motivation in the MID Task (*p* = 0.028; *p* = 0.017). For feedback gain, activation of the right insula was increased in controls, and activation correlated with gain expectancy and satisfaction about gain. Subjective value is not detached from reward parameters, but is modulated from expectancy and reward by the insula. The underlying neural mechanisms are a fundamental target point for treatments, interventions, and cognitive behavioral therapy.

## Introduction

The US National Institute of Mental Health has developed the Research Domain Criteria (RDoC) approach to explore the underlying biological causes of mental disorders (1). For this purpose, a research framework has been established to link and integrate current clinical syndromes with basic biological and behavioral components. The goal seeks to understand mental functioning in continuous valid dimensions ranging from functional to pathological. The RDoC framework consists of five predefined domains of functioning. The negative and the positive valence systems cover loss and reward constructs, such as loss, reward anticipation, reward prediction error, habit, and reward valuation including delay (2). Constructs are analyzed in a multi-dimensional approach comprising genes, circuits, observed behaviors, and self-reports (3).

Motivation is the energizing of behavior in pursuit of a goal, and obtaining goals or basic needs appropriately is rewarding (4). Thus, reward processes and motivation are closely linked. For example, reward contingency is used in current cognitive/behavioral treatments for schizophrenia and addiction to modify behavioral deficits or excess in motivation (5). Reward-related symptoms appear in the diagnostic criteria for multiple disorders and are thus transdiagnostic in nature (6). Reward processing abnormalities are a key component in the development and manifestation of a wide range of psychopathologies, including addictions (5). For instance, cannabis use is associated with amotivational syndrome (6). The effect of cannabis use on neural reward processing has been investigated intensively with the well-established reward paradigm of the monetary incentive delay (MID) task (7). The results display alterations in reward-related neural functioning (8, 9). For example, cannabis users showed hypoactivity in the left insula cortex in response to loss and loss avoidance outcomes (8). Cannabis and nicotine are often used at the same time and altered reward functioning could also be verified for nicotine use (10, 11). According to previous findings, nicotine use does not affect amotivational syndrome (6). The co-use of cannabis and nicotine and mutual reinforcement is a critical matter of prevention and public health (10).

Physical reward parameters or reward cue properties, such as magnitude, cannot precisely define subjective reward value (12). Value is an internal component of reward and is represented in subjective preferences, such as individual needs or emotional valence (5, 13). Representations are generated by brain mechanisms mediated by dopamine neurons in the substantia nigra and VTA, and phasic dopamine responses increase with the expected reward value (13). Nevertheless, the assessment of unobservable subjective value is a central challenge in reward research (5). One way to resolving this issue is to ask people how rewarding they find something and to compare choices between objectively equal rewards (5, 13).

Within the RDoC approach, genetic variants are among the units of analysis (3). The Val158Met polymorphism of the gene for catechol-*O*-methyltransferase (COMT) leads to valine to methionine exchange at position 158 of the protein (14). Homozygotes for the 158Met allele exhibit 35–50% lower brain COMT activity than homozygotes for 158Val with higher extrasynaptic dopamine levels, while heterozygotes show an intermediate enzyme function (15). Wichers et al. investigated the effect of the Val158Met polymorphism on the ability to experience reward in daily life (16). Homozygotes for 158Met generated almost similar amounts of subjective wellbeing from a “bit pleasant” daily life experience as 158Val homozygotes did from a “very pleasant” experience. The ability to experience reward increased with the number of “Met” alleles. Despite the genetic differences in subjective reward processing, the daily life experiences in the study of Wichers et al. were neither comparable reward cues nor objectively equal rewards. Reward experience was operationalized as the effect of event appraisal on positive affect and associations between COMT genotype, event appraisal, and positive affect were examined with regression analysis. In addition, reward sensitivity was also measured with self-reports, as well as behavioral tasks (17). Increased reward sensitivity was predictive of substance use, substance use disorders, greater cravings, and positive affective responses in alcohol cue reactivity paradigms. In this context, genetic modulation of reward sensitivity *via* dopamine transmission may be of special interest to understand individual differences (17, 18).

The aim of the present pilot study is to investigate reward processing for the first time dimensionally in the context of the RDoC approach, rather than focusing on mental disorder categories only (3). Reward processing is a transdiagnostic functioning domain of the RDoC. Due to prototypical abnormalities, addictions are especially applicable for the investigation of reward processing and the focus is on the potential effects of nicotine and cannabis on amotivational syndrome and dimensions of subject reward processing, such as motivation. Subjective reward processing differs between Val158Met genotypes for incomparable daily life experiences and was assessed with delay after the experiences. In the present study, this issue was methodically resolved with visual analog scales (VAS) or self-reports, comparable reward cues, and a constant reinforcement rate. We hypothesized that homozygotes for 158Met would be more satisfied with the achieved gain in the MID Task than individuals with the other genotypes.

## Materials and methods

### Participants

Thirteen inpatients with cannabis dependency and 13 volunteers without cannabis abuse participated in the study. The sample size was set to a minimum of 24 participants to anticipate results for the main study (19, 20). All participants were male subjects, right-handed smokers of European ancestry. The participants had been instructed to discontinue cigarette smoking for at least 2 h before the study and were interviewed and assessed by a clinical psychologist. The diagnosis of the cannabis-dependent inpatients was confirmed with the International Diagnostic Checklists for DSM-IV and axis I or II disorders other than nicotine dependence were excluded for all participants (21, 22). A urine drug test was conducted to control for the use of cannabis and other drugs (23). Any history of the abuse of other drugs, psychiatric, neurological or chronic diseases, head trauma, loss of consciousness, impaired vision, and the use of medication for volunteers were exclusion criteria. Nicotine dependence was assessed with the Fagerström Test (FTNA) and the participants were matched on nicotine dependence, age, verbal intelligence, and years of education (24). The groups did not differ in any dimension of the controlled variables (*P* ≥ 0.064).

Cannabis consumption was assessed with self-report questionnaires and the European Addiction Severity Index questionnaire (EuropASI) (25). Only cannabis-dependent inpatients with at least 4 days of abstinence and without withdrawal symptoms were included. The mean age of initial use was 16 years (SD = 2), the mean cannabis use was 8 years (SD = 6), and 12 grams per week (SD = 5). The mean abstinence was 24 days (SD = 24).

### Genotyping

DNA was extracted from the EDTA anticoagulated blood samples of all participants. A fragment containing the COMT Val158Met polymorphism (dbSNP: rs4680) was amplified with polymerase chain reaction (PCR). Sanger sequencing of this fragment was conducted commercially (26). Analyses of the sequences were performed by eye aided by the software Lasergene (27). Table 1 summarizes the genotypes of both study groups. Hardy Weinberg equilibrium was fulfilled for all study groups.

**TABLE 1:** *COMT* genotypes of individuals who smoke (*N* = 13) and are cannabis dependent (*N* = 13).

COMT genotype	Nicotine N (percent)	Cannabis N (percent)	Σ
Val/Val	7 (54)	4 (31)	11
Val/Met	4 (31)	5 (38)	9
Met/Met	2 (15)	4 (31)	6
Σ	13 (100)	13 (100)	26

### Reward reaction task and subjective reward processing

**FIGURE 1:**
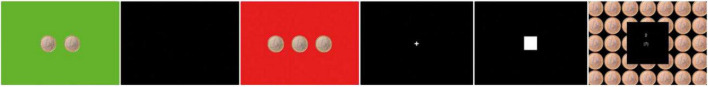
Cues for gain (green), neutral (black), loss (red) 250 ms, delay (cross) 2.25–2.75 s target (white square), and feedback with outcome 1650 ms.

The reward reaction task was based on the monetary incentive delay (MID) task (7). Participants had to react to monetary reward cues with the push of a button (Figure 1 and Supplementary material).

The amount of gain and loss varied between 1, 2, and 3 Euros corresponding to the number of 1 Euro coins on the picture. The participants started with a credit of 5 Euros. They completed one practice run and two test runs. In the test runs, the target presentation was adapted to the individual reaction rates and an average hit rate of 50% was predefined. The participants were informed that they would be paid the higher outcome of one of the two test runs. Subjective reward processing was assessed with visual analog scales (VAS) correspondingly to the study of Wrase et al. (28). Before the reward reaction task, the participants were asked to rate their motivation and gain expectancy. After the task, they rated their effort for a gain of 3 Euros compared with 2 Euros and 1 Euro, their fear of a loss of 3 Euros compared with 2 Euros and 1 Euro, and their satisfaction with the achieved gain. Finally, the participants completed the items of the personality trait Reward Dependence of the Temperament and Character Inventory (TCI) (29).

### fMRI data acquisition

fMRI data acquisition was performed on a 3 T magnetic resonance scanner (Magnetom VISION Siemens^®^) with a circularly polarized standard head coil (CP-Headcoil). For anatomical reference, a 3 D Magnetization Prepared Rapid Gradient Echo (MPRAGE) data set was acquired with the following parameters: TR = 9.7 ms, TE = 4 ms, flip angle 12°, matrix = 256 × 256, and voxel size 1 mm × 1 mm × 1mm. For functional scans, a gradient-echo echo-planar imaging (GE-EPI) sequence was conducted with the parameters TR = 1.9 s, TE = 30 ms, flip angle = 90°, matrix = 64 × 64, and voxel size = 3.8 mm × 3.8 mm × 3.3 mm. FMRI volume acquisition was time-locked to the offset of the cues.

### fMRI data analysis

fMRI data were analyzed for BOLD responses to reward anticipation and feedback. Data analysis was conducted with SPM8 (30). Voxel time series were interpolated to adjust non-simultaneous slice acquisition within each volume. Motion artifacts were corrected. Head movements were below 3 mm in translation and 3° in rotation from one volume acquisition to the next. The anatomical images were coregistered with the mean functional images. For normalization, the coregistered image was first spatially normalized to the standard template provided by the Montreal Neurological Institute (MNI-Template), and the obtained normalization parameters were then applied to all functional images. Voxel time series were smoothed with a Gaussian kernel (FWHM = 8 mm).

In the first-level analysis, a statistical model with all conditions was computed for each participant according to the general linear model approach (31, 32). Gain and loss were contrasted with the neutral conditions, e.g., “anticipation of gain” vs. “no anticipation.” Contrasts were calculated as t-statistic for each voxel.

In the second-level analysis, contrasts within the reward anticipation and feedback conditions were calculated for each group with a one-sample *t*-test at a significance level of *P* < 0.005 and a cluster threshold of *k* > 5. One-sample *t*-tests were FDR-corrected (33). Contrasts between cannabis-dependent inpatients and the control group were calculated for each condition with a two-sample *t*-test at a significance level of *P* < 0.005 and a cluster threshold of *k* > 9. The activated brain areas were determined on the basis of the coordinates of Hägele et al. (34). Regions of interest (ROIs; radius 5 mm) were sphere shaped and centered upon the peak voxel within each area of interest. The ROIs beta-values for each condition were extracted and converted into percent signal change using the Marseille Region of Interest Toolbox (35) software package (36).

### Behavioral data analysis

With *t*-tests and multivariate analyses of variance, subjective reward processing, Reward dependence, and reaction times were analyzed for group differences between cannabis-dependent inpatients and the control group. Subsequently, these variables were analyzed for Pearson’s correlations with activated brain region and the number of met alleles. Bonferroni corrections were conducted to control the family-wise error rate. With Kruskal–Wallis H tests, group differences for nicotine use and subjective reward processing were analyzed between genotypes. Behavioral data analysis was calculated with the software package SPSS 22.0 (SPSS Inc., Chicago, IL, USA). Results with a *p*-value below 0.05 were considered statistically significant.

## Results

### Reward anticipation and feedback

**FIGURE 2:**
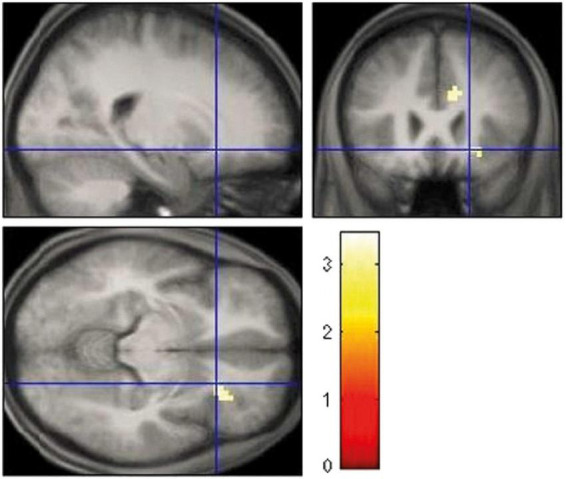
Increased right insula activation for feedback gain in smoking vs. cannabis-dependent individuals.

Table 2 gives an overview of the brain regions revealing the main effects of reward anticipation and feedback. During feedback, no loss, activation of the right inferior frontal gyrus was increased in individuals who smoke (*x* = 51; *y* = 32; *z* = 1). Table 3 contains the brain regions with activation differences between the groups. Individuals who smoke showed an increased activation of the right insula for the anticipation of gain vs. loss (*x* = 60; *y* = 5; *z* = 4), and for feedback gain (*x* = 21; *y* = 23; *z* = −8; Figure 2).

**TABLE 2:** Activated brain regions through reward anticipation and feedback in individuals who smoke (*N* = 13) and are cannabis dependent (*N* = 13).

Contrast	Study group (*N* = 13)	Brain region	x	y	z	t	*P*
Anticipation gain vs. loss	Nicotine	R insula	39	−7	7	4.32	0.001
	Cannabis	R anterior cingulate cortex (ACC)	9	29	28	3.45	0.005
Anticipation loss vs. gain	Nicotine	L inferior frontal gyrus (IFG)	−54	17	31	4.50	0.001
	Cannabis	R primary somatosensory cortex	33	−40	58	3.76	0.001
Feedback gain	Nicotine	L postcentral gyrus	−60	−22	49	6.21	0.001
	Cannabis	R middle temporal gyrus (MTG)	57	−28	−2	7.15	0.001
		R superior temporal gyrus (STG)	60	−34	16	5.69	0.001
		R inferior frontal gyrus (IFG)	48	20	16	4.88	0.001
Feedback loss	Nicotine	R middle frontal gyrus (MFG)	24	56	−8	6.75	0.001
		R inferior frontal gyrus (IFG)	51	32	1	5.79	0.001
		R insula	27	23	−11	4.63	0.001
	Cannabis	R middle temporal gyrus (MTG)	60	−49	10	5.43	0.001
		R inferior frontal gyrus (IFG)	60	8	10	5.18	0.001
		R cingulum	12	−34	46	4.36	0.001

**TABLE 3:** Contrasts with differently activated brain regions (*P* ≤ 0.001).

Contrast	Group comparison	Brain region	x	y	Z	t
Anticipation gain vs. loss	Nicotine > Cannabis	R insula	60	5	4	3.96
Anticipation loss vs. gain	Nicotine > Cannabis	L inferior frontal gyrus (IFG)	−48	50	1	3.96
Feedback gain	Nicotine > Cannabis	R insula	21	23	−8	3.46
Feedback loss	Nicotine > Cannabis	R inferior frontal gyrus (IFG)	63	11	16	4.37
		L inferior frontal gyrus (IFG)	−48	14	13	4.02
		L putamen	−30	5	4	3.91
		R insula	30	23	1	3.65

### Subjective reward processing and catechol-*O*-methyltransferase genotype

Individuals who smoke and cannabis-dependent inpatients did not differ significantly in subjective reward processing or reward dependence (all *P* > 0.28; Supplementary material).

A Kruskal–Wallis H test showed nominal differences in cigarettes per day χ2(2) = 7.118, *p* = 0.028 and motivation χ2(2) = 8.192, *p* = 0.017 between COMT genotypes with a mean rank of 11.00 for Val/Val, 11.72 for Val/Met, and 20.75 for Met/Met and 11.86 for Val/Val, 19.06 for Val/Met, and 8.17 for Met/Met. There were statistically significant correlations for the anticipation of gain vs. loss in cannabis dependents, for feedback loss in smokers, and for feedback gain in individuals who smoke and cannabis-dependent inpatients (Supplementary material). For feedback gain, activation of the insula correlated negatively with gain expectance and positively with satisfaction with the achieved gain in individuals who smoke and cannabis-dependent inpatients (*p* < 0.004; *p* < 0.014).

## Discussion

In the present pilot study, we investigated subject reward processing and the COMT Val158Met polymorphism in abstinent cannabis-dependent inpatients and individuals who smoke with the well-established reward paradigm of the MID Task and found the commonly activated brain regions for this task (Table 4; 37). The aim was to take reward processing into account dimensionally across different forms of addiction, rather than only focusing on overarching mental disorder categories. The study focused on the application of the RDoC as well as the feasibility to assess subject reward processing.

**TABLE 4:** The main findings for the MID Task, the COMT Val158Met polymorphism, and its implications.

• **For feedback gain:** Activation of the right insula was increased in controls and activation correlated with gain expectancy and satisfaction about gain.
• Subjective value is not detached from reward parameters, but is modulated from expectancy and reward by the insula
• **COMT Val158Met:** Genotypes were nominally associated with differences in cigarettes smoked per day and motivation in the MID Task.
• Homozygotes for 158Met may have less negative subjective effects of nicotine and thus smoke more.

Subjective reward processing did not differ between nicotine and cannabis in any dimension. Nonetheless, the results suggest that the most likely dimensions for differences in a larger sample are effort gain and satisfaction about gain. Because of the small sample size and other methodical limitations, the claim was not to draw a statistical inference for genetic neuroimaging and the sample size was set to a minimum of 24 participants. The sample size should not have been set in terms of cannabis and nicotine use, but the COMT genotype. In the total sample, homozygotes for 158Met were not more satisfied with the achieved gain than carriers of the other genotypes, which is probably due to the sample size. However, genotypes nominally differ for cigarettes per day and motivation with the highest number of cigarettes and the lowest motivation rank for homozygotes of 158Met. Tentatively, one might assume that homozygotes for 158Met may have less negative subjective effects of nicotine and thus smoke more (38). Reduced motivation in homozygotes of 158Met has previously been found in men of a Swedish sample (39). For individuals who smoke, cigarettes per day and motivation correlated negatively. The profile of nicotine changes from stimulant to sedative with increasing dosage (40). The present correlation can thus be attributed to the “Nesbitt’s paradox” (38). Cannabis also affects motivation (41). Therefore, cigarettes and motivation were putatively not associated with cannabis dependence. In individuals who smoke, cigarette use correlated negatively and motivation positively with the activation of the right inferior frontal gyrus during feedback no loss. The right inferior frontal gyrus has been assigned to self-control, reward prediction errors, and sensation seeking and high sensation seekers are less sensitive to punishment (42–45). Higher cigarette use may hence imply lower self-control and response inhibition. Moreover, the present results suggest that motivation may be high precisely when predictions do not occur regardless of negative consequences.

For anticipation gain vs. loss, individuals who smoke had increased activation of the right insula compared with cannabis dependents. Acute THC increases perfusion in the insula with stronger increases for Val/Met heterozygotes (46). In anticipating rewards, insula activation is associated with motivational salience and sensitivity to reward magnitude (47, 48). Acute nicotine sensitizes and chronic smoking enhances the right insular response to gain and loss anticipation (11, 49). In contrast, activation of the left insular is reduced through acute cannabidiol and correlates with the salience of reward anticipation (48). The different contrast of anticipation gain vs. loss in the present study rather than gain vs. neutral cues in previous studies could be due to the diminished success probability of 50% (37). The current results suggest an overall desensitizing effect of the co-use of nicotine and cannabis on the insula and a decrease in salience for reward anticipation.

For feedback gain, individuals who smoke had increased activation of the right insula compared with cannabis-dependent inpatients. For reward feedback, the insula is involved in affective responding (37). The right anterior insula mediates interoceptive awareness (50). It integrates affective value with bodily states for reward-related adaptive behavior (51, 52). In the total sample, the activation of the right insula during feedback gain correlated negatively with gain expectancy and positively with satisfaction about the achieved gain. This finding indicates that the right insula interconnects gain expectancy with the evaluation of the reward-related performance and outcome independent of the substance. Additionally, the gain of the second test run correlated negatively with gain expectancy and positively with satisfaction. Higher gain expectancy at the beginning reduces reinforcement and thus motivation over the course of the MID Task, which impairs task monitoring and performance. Moderate or lower expectations have the opposite effect and therefore increase satisfaction with the outcome. The insula and interoceptive awareness are also crucial for drug cravings (53–55). The insula elicits conscious interoceptive urges, such as drug craving in response to rewarding and drug stimuli, respectively. For cannabis-dependent inpatients, gain expectancy correlated negatively with satisfaction and abstinence. Satisfaction correlated positively with consumption. These associations suggest a sensitizing effect of cannabis on subjective reward processing with an increase in gain expectancy and satisfaction. The dose-dependent increase in satisfaction is putatively due to the acute psychoactive effect of cannabis (56). Acute subjective changes in relaxation and perception caused by cannabis are also related to its effect on the insula (46). Positive subjective drug expectancies impair decision-making of addicted patients (57). The impairment consists of an overreliance on habits at the expense of goal-directed behavior and may be particularly symptomatic for early abstinence. As subjects had no experience with the MID Task (7), rationally, they should have quoted a middle gain expectancy. The negative correlation between abstinence and gain expectancy confirms drug-associated habitual biases during early abstinence. Moreover, insular functioning is highly dependent upon dopamine transmission and aberrant activation indicates impaired cue processing (58). Higher-than-predicted rewards generate positive prediction errors and elicit brief dopamine activations, whereas lower-than-predicted rewards generate negative prediction errors and induce decreases in activity (12). Accurately predicted rewards do not change the activity. For cannabis-dependent inpatients, gain expectancy correlated negatively with the activation of the right anterior cingulate cortex during the anticipation of gain vs. loss. The anterior cingulate cortex is involved in error monitoring and is affected by nicotine and cannabis (59, 60). For error monitoring, the expectation about an action is compared with the outcome and a deviation elicits the correction of behavioral responses (61). Any expectancy such as the current gain expectancies distract *per se* from task performance. Higher gain expectancies may additionally shift and bias attention toward cues for monetary gain under the neglect of losses. In consideration of the diminished success probability of 50%, however, it is just as important to avoid losses, as it is to gain money. Increased activity of the anterior cingulate cortex may suggest that cannabis-dependent inpatients with lower gain expectancy differentiated more effectively between gain and loss cues and thus, equally focused on and endeavored to avoid losses.

At first glance, the assessment of subjective reward processing seems exceptionally challenging. Subjective reward values such as preferences, needs, or emotional valence are unobservable, highly individual, and thus indefinable by physical reward parameters (12). In the present study, this issue was methodically resolved with visual analog scales (VAS) or self-reports, comparable reward cues, and a constant reinforcement rate. The variable gain instead of a predefined gain for all subjects was a limitation of the study. Nevertheless, the results suggest that subjective value is not completely detached from reward parameters, but a quotient between value and parameter: previous gain expectancies namely modulate the subsequent reward evaluation. Abnormalities in reward processing are prototypical for addictions and comprise a sensitizing effect of cannabis on subjective reward processing with an increase in gain expectancy and satisfaction according to the present results. Higher gain expectancies, whether cannabis-induced or not, reduce reinforcement, motivation, and satisfaction of the outcome and the insula might function as an inverse balance between expectation and satisfaction. The COMT Val158Met polymorphism might influence motivation. Any expectation distracts from task performance by affecting error monitoring and response correction of reward-related behavior negatively. Addiction research suggests that expectations or biases might be traced back to the overreliance on habits at the expense of goal-directed behavior. The same bias and overreliance on habits were reported for non-clinical obsessive-compulsive symptoms (62). Obtaining goals or basic needs in an appropriate manner is rewarding, and the pursuit of a goal defines motivation (4). Therefore, cognitive biases and the underlying neural mechanisms of error monitoring, response correction, and goal attainment might be the most fundamental target points of mental functioning and treatment.

In the RDoC framework, subjective reward processing can be assigned to the domain of the negative and positive valence systems. The COMT Val158Met polymorphism, the reward system including the insula, performance in the MID Task, and self-reports were utilized as units of analysis. Subjective value is not detached from reward parameters but is a modulated from expectancy and reward by the insula. The insula is crucial for the interconnection of expectance and evaluation and aberrant activation might imply incongruence. Cognitive biases and the underlying neural mechanisms are thus the most fundamental target point for treatments, interventions, and cognitive behavioral therapy in further research. Examples of initial approaches are Cognitive Bias Modification and Emotional Bias Modification (63–65). These approaches are likely to be intensively extended and to be future of RDoC diagnostics and predominantly neural psychotherapy.

## Data availability statement

The original contributions presented in this study are included in the article/Supplementary material, further inquiries can be directed to the corresponding author.

## Ethics statement

This study was reviewed and approved by Ethics Committee at the University of Duisburg-Essen. The patients/participants provided their written informed consent to participate in this study.

## Author contributions

NR and NS designed the study. NR was involved in the data acquisition and wrote the manuscript. AH was involved in genotyping. UB, IT, AH, and NS revised the manuscript. All authors read and approved the manuscript.

## References

[1] Insel T Cuthbert B Garvey M Heinsse R Pine DS Quinn K Research domain criteria (RDoC): Toward a new classification framework for research on mental disorders. *Am J Psychiatry.* (2010) 167:748–51. 10.1176/appi.ajp.2010.09091379 20595427

[2] Cuthbert BN Insel TR. Toward the future of psychiatric diagnosis: the seven pillars of RDoC. *BMC Med.* (2013) 11:126. 10.1186/1741-7015-11-126 23672542 PMC3653747

[3] National Institute of Mental Health. (2022). Available online at: https://www.nimh.nih.gov/search-nimh?q=RDoC (accessed June 26, 2022).

[4] Simpson EH Balsam PD. The Behavioral Neuroscience of Motivation: An Overview of Concepts, Measures, and Translational Applications. *Curr Top Behav Neurosci.* (2016) 27:1–12. 10.1007/7854_2015_402 26602246 PMC4864984

[5] Zald DH Treadway MT. Reward processing, neuroeconomics, and psychopathology. *Annu Rev Clin Psychol.* (2017) 13:471–95. 10.1146/annurev-clinpsy-032816-044957 28301764 PMC5958615

[6] Lac A Luk JW. Testing the amotivational syndrome: marijuana use longitudinally predicts lower self-efficacy even after controlling for demographics, personality, and alcohol and cigarette use. *Prev Sci.* (2018) 19:117–26. 10.1007/s11121-017-0811-3 28620722 PMC5732901

[7] Knutson B Adams CM Fong GW Hommer D. Anticipation of increasing monetary reward selectively recruits nucleus accumbens. *J Neurosci.* (2001) 21:1–5. 10.1523/JNEUROSCI.21-16-j0002.2001 11459880 PMC6763187

[8] Nestor L Hester R Garavan H. Increased ventral striatal BOLD activity during non-drug reward anticipation in cannabis users. *Neuroimage.* (2010) 49:1133–43. 10.1016/j.neuroimage.2009.07.022 19631753 PMC2764826

[9] Jager G Block RI Luijten M Ramsey NF. Tentative evidence for striatal hyperactivity in adolescent cannabis-using boys: a cross-sectional multicenter fMRI study. *J Psychoactive Drugs.* (2013) 45:156–67. 10.1080/02791072.2013.785837 23909003 PMC3733475

[10] Jayakumar N Chaiton M Goodwin R Schwartz R O’Connor S Kaufman P. Co-use and mixing tobacco with cannabis among ontario adults. *Nicotine Tob Res.* (2021) 23:171–8. 10.1093/ntr/ntz238 31867605 PMC7789941

[11] Nestor LJ McCabe E Jones J Clancy L Garavan H. Smokers and ex-smokers have shared differences in the neural substrates for potential monetary gains and losses. *Addict Biol.* (2018) 23:369–78. 10.1111/adb.12484 27943592

[12] Schultz W. Dopamine reward prediction-error signalling: a two-component response. *Nat Rev Neurosci.* (2016) 17:183–95. 10.1038/nrn.2015.26 26865020 PMC5549862

[13] Schultz W. Neuronal reward and decision signals: from theories to data. *Physiol Rev.* (2015) 95:853–951. 10.1152/physrev.00023.2014 26109341 PMC4491543

[14] Lachman HM Papolos DF Saito T Yu YM Szumlanski CL Weinshilboum RM. Human catechol-o-methyltransferase pharmacogenetics: description of a functional polymorphism and its potential application to neuropsychiatric disorders. *Pharmacogenetics.* (1996) 6:243–50. 10.1097/00008571-199606000-00007 8807664

[15] Chen J Lipska BK Halim N Ma QD Matsumoto M Melhem S Functional analysis of genetic variation in catechol-o-methyltransferase (COMT): effects on mRNA, protein, and enzyme activity in postmortem human brain. *Am J Hum Genet.* (2004) 75:807–21. 10.1086/425589 15457404 PMC1182110

[16] Wichers M Aguilera M Kenis G Krabbendam L Myin-Germeys I Jacobs N The catechol-O-methyl transferase Val158Met polymorphism and experience of reward in the flow of daily life. *Neuropsychopharmacology.* (2008) 33:3030–6. 10.1038/sj.npp.1301520 17687265

[17] Nusslock R Alloy LB. Reward processing and mood-related symptoms: An RDoC and translational neuroscience perspective. *J Affect Disord.* (2017) 216:3–16. 10.1016/j.jad.2017.02.001 28237133 PMC6661152

[18] Baskin-Sommers AR Foti D. Abnormal reward functioning across substance use disorders and major depressive disorder: Considering reward as a transdiagnostic mechanism. *Int J Psychophysiol.* (2015) 98:227–39. 10.1016/j.ijpsycho.2015.01.011 25655926

[19] Whitehead AL Julious SA Cooper CL Campbell MJ. Estimating the sample size for a pilot randomised trial to minimise the overall trial sample size for the external pilot and main trial for a continuous outcome variable. *Stat Methods Med Res.* (2016) 25:1057–73. 10.1177/0962280215588241 26092476 PMC4876429

[20] Julious SA. Sample size of 12 per group rule of thumb for a pilot study. *Pharmaceut Stat.* (2005) 4:287–91. 10.1002/pst.185

[21] Hiller W Zaudig M Mombour W. *Internationale Diagnosen Checklisten (IDCL) für DSM-IV.* Göttingen: Hans Huber (1997). 10.1007/978-3-642-13018-2_16

[22] Bronisch T Hiller W Mombour W Zaudig M. *Internationale Diagnosen Checkliste für Persönlichkeitsstörungen.* Göttingen: Hans Huber (1995). 10.1007/978-3-7091-6767-0_24

[23] Nal Von Minden. (2022). Available online at: https://www.nal-vonminden.com (accessed June 26, 2022).

[24] Fagerström K-O. Measuring the degree of physical dependency to tobacco smoking with references to individualization of treatment. *Addict Behav.* (1978) 3:235–41. 10.1016/0306-4603(78)90024-2 735910

[25] Gsellhofer B Fahrner EM. *EuropASI (German Version of the Addiction Severity Index). Reproduced Manuscript of the American Original in the version of 1992.* Munich: IFT Institute for Therapy Research (1994).

[26] Microsynth/Seqlab. (2022). Available online at: https://www.microsynth.com. (accessed June 26, 2022).

[27] DNASTAR. (2022). Available online at: http://www.dnastar.com. (accessed June 26, 2022).

[28] Wrase J Schlagenhauf F Kienast T Wüstenberg T Bermpohl F Kahnt T Dysfunction of reward processing correlates with alcohol craving in detoxified alcoholics. *Neuroimage.* (2007) 35:787–94. 10.1016/j.neuroimage.2006.11.043 17291784

[29] Cloninger RC. *The temperament and character inventory (TCI): A guide to its development and use.* Seattle: Washington University (1994).

[30] Wellcome Department of Neuroimaging. (2022). Available online at: http://www.fil.ion.ucl.ac.uk/spm (accessed June 26, 2022).

[31] Friston K Holmes A Worsley K Poline J Frith C Frackowiak J Statistical parametric maps in functional imaging: a general linear approach. *Hum Brain Map.* (1995) 2:189–210.

[32] Friston K Fletcher P Josephs O Holmes A Rugg M Turner R. Event-related fMRI: characterizing differential responses. *Neuroimage.* (1998) 7:30–40. 10.1006/nimg.1997.0306 9500830

[33] Genovese CR Lazar NA Nichols T. Thresholding of statistical maps in functional neuroimaging using the false discovery rate. *Neuroimage.* (2002) 4:870–8. 10.1006/nimg.2001.1037 11906227

[34] Hägele C Schlagenhauf F Rapp M Sterzer P Beck A Bermpohl F Dimensional psychiatry: reward dysfunction and depressive mood across psychiatric disorders. *Psychopharmacology.* (2015) 232:331–41. 10.1007/s00213-014-3662-7 24973896 PMC4297301

[35] MarsBaR. (2022). Available online at: http://marsbar.sourceforge.net/ (accessed June 26, 2022).

[36] Brett M Anton J Valabregue R Poline J. Region of interest analysis using an SPM toolbox. *Proceedings of the 8th International Conference on Functional Mapping of the Human Brain.* Sendai (2002).

[37] Dugré JR Dumais A Bitar N Potvin S. Loss anticipation and outcome during the Monetary Incentive Delay Task: a neuroimaging systematic review and meta-analysis. *Peer J.* (2018) 6:e4749. 10.7717/peerj.4749 29761060 PMC5949205

[38] Herman AI Jatlow PI Gelernter J Listman JB Sofuoglu M. COMT Val158Met modulates subjective responses to intravenous nicotine and cognitive performance in abstinent smokers. *Pharmacogenomics J.* (2013) 13:490–7. 10.1038/tpj.2013.1 23459442 PMC3675163

[39] Åberg E Fandiño-Losada A Sjöholm LK Forsell Y Lavebratt C. The functional Val158Met polymorphism in catechol-O-methyltransferase (COMT) is associated with depression and motivation in men from a Swedish population-based study. *J Affect Disord.* (2011) 129:158–66. 10.1016/j.jad.2010.08.009 20828831

[40] Parrott AC. Nesbitt’s Paradox resolved? Stress and arousal modulation during cigarette smoking. *Addiction.* (1998) 93:27–39. 10.1046/j.1360-0443.1998.931274.x 9624709

[41] Volkow ND Swanson JM Evins AE DeLisi LE Meier MH Gonzalez R Effects of cannabis use on human behavior, including cognition, motivation, and psychosis: a review. *JAMA Psychiatry.* (2016) 73:292–7. 10.1001/jamapsychiatry.2015.3278 26842658

[42] Romaniuk L Sandu AL Waiter GD McNeil CJ Xueyi S Harris MA The neurobiology of personal control during reward learning and its relationship to mood. *Biol Psychiatry Cogn Neurosci Neuroimaging.* (2019) 4:190–9. 10.1016/j.bpsc.2018.09.015 30470583 PMC6374985

[43] Cao Z Bennett M Orr C Icke I Banaschewski T Barker GJ IMAGEN consortium. mapping adolescent reward anticipation, receipt, and prediction error during the monetary incentive delay task. *Hum Brain Mapp.* (2019) 40:262–83. 10.1002/hbm.24370 30240509 PMC6865381

[44] Muhlert N Boy F Lawrence DA. Risk taking, response inhibition and the right inferior frontal gyrus. *J Neurol Neurosurg.* (2015) 86:8.

[45] Kruschwitz JD Simmons AN Flagan T Paulus MP. Nothing to lose: processing blindness to potential losses drives thrill and adventure seekers. *Neuroimage.* (2012) 59:2850–9. 10.1016/j.neuroimage.2011.09.048 21982930 PMC3256346

[46] Bossong MG van Hell HH Schubart CD van Saane W Iseger TA Jager G. Acute effects of Δ9-tetrahydrocannabinol (THC) on resting state brain function and their modulation by COMT genotype. *Eur Neuropsychopharmacol.* (2019) 29:766–76. 10.1016/j.euroneuro.2019.03.010 30975584

[47] Dhingra I Zhang S Zhornitsky S Le TM Wang W Chao HH The effects of age on reward magnitude processing in the monetary incentive delay task. *Neuroimage.* (2020) 207:116368. 10.1016/j.neuroimage.2019.116368 31743790 PMC7463276

[48] Wilson R Bossong MG Appiah-Kusi E Petros N Brammer M Perez J Cannabidiol attenuates insular dysfunction during motivational salience processing in subjects at clinical high risk for psychosis. *Transl Psychiatry.* (2019) 9:203. 10.1038/s41398-019-0534-2 31439831 PMC6706374

[49] Moran LV Stoeckel LE Wang K Caine CE Villafuerte R Calderon V Nicotine increases activation to anticipatory valence cues in anterior insula and striatum. *Nicotine Tob Res.* (2018) 7:851–8. 10.1093/ntr/ntx217 29059451 PMC5991218

[50] Craig AD. How do you feel–now? The anterior insula and human awareness. *Nat Rev Neurosci.* (2009) 10:59–70. 10.1038/nrn2555 19096369

[51] Critchley HD Harrison NA. Visceral influences on brain and behaviour. *Neuron.* (2013) 77:624–38. 10.1016/j.neuron.2013.02.008 23439117

[52] Craig AD. How do you feel? Interoception: the sense of the physiological condition of the body. *Nat Rev Neurosci.* (2002) 3:655–66. 10.1038/nrn894 12154366

[53] Gray MA Critchley HD. Interoceptive basis to craving. *Neuron.* (2007) 54:183–6. 10.1016/j.neuron.2007.03.024 17442239 PMC2259270

[54] Naqvi NH Rudrauf D Damasio H Bechara A. Damage to the insula disrupts addiction to cigarette smoking. *Science.* (2007) 315:531–4. 10.1126/science.1135926 17255515 PMC3698854

[55] Paulus MP. Decision-making dysfunctions in psychiatry-altered homeostatic processing? *Science.* (2007) 318:602–6. 10.1126/science.1142997 17962553

[56] Karila L Roux P Rolland B Benyamina A Reynaud M Aubin HJ Acute and long-term effects of cannabis use: a review. *Curr Pharm Des.* (2014) 20:4112–8. 10.2174/13816128113199990620 24001294

[57] Sebold M Nebe S Garbusow M Guggenmos M Schad DJ Beck A When habits are dangerous: alcohol expectancies and habitual decision making predict relapse in alcohol dependence. *Biol Psychiatry.* (2017) 82:847–56. 10.1016/j.biopsych.2017.04.019 28673442

[58] de Leeuw M Kahn RS Vink M. Fronto-striatal dysfunction during reward processing in unaffected siblings of schizophrenia patients. *Schizophr Bull.* (2015) 41:94–103. 10.1093/schbul/sbu153 25368371 PMC4266310

[59] Nestor LJ McCabe E Jones J Clancy L Garavan H. Shared and divergent neural reactivity to non-drug operant response outcomes in current smokers and ex-smokers. *Brain Res.* (2018) 1680:54–61. 10.1016/j.brainres.2017.12.003 29242147

[60] Aloi J Meffert H White SF Blair KS Hwang S Tyler PM Differentia dysfunctions related to alcohol and cannabis use disorder symptoms in reward and error-processing neuro-circuitries in adolescents. *Dev Cogn Neurosci.* (2019) 36:100618. 10.1016/j.dcn.2019.100618 30710868 PMC6613939

[61] Luu P Pederson SM. The anterior cingulate cortex: Regulating actions in context. In: Posner MI editor. *Cognitive neuroscience of attention.* New York, NY: Guilford Press (2004).

[62] Snorrason I Lee HJ de Wit S Woods DW. Are nonclinical obsessive-compulsive symptoms associated with bias toward habits? *Psychiatry Res.* (2016) 241:221–3. 10.1016/j.psychres.2016.04.067 27183107

[63] Batschelet HM Stein M Tschuemperlin RM Soravia LM Moggi F. Alcohol-Specific computerized interventions to alter cognitive biases: a systematic review of effects on experimental tasks, drinking behavior, and neuronal activation. *Front Psychiatry.* (2020) 10:871. 10.3389/fpsyt.2019.00871 31998146 PMC6970199

[64] Jones EB Sharpe L. Cognitive bias modification: A review of meta-analyses. *J Affect Disord.* (2017) 223:175–83. 10.1016/j.jad.2017.07.034 28759865

[65] Hirsch CR Krahé C Whyte J Bridge L Loizou S Norton S Effects of modifying interpretation bias on transdiagnostic repetitive negative thinking. *J Consult Clin Psychol.* (2020) 88:226–39. 10.1037/ccp0000455 32068424

