# Supplementary material for: Subjective reward processing and catechol-O- methyltransferase Val158Met polymorphism as potential research domain criteria in addiction: A pilot study
Source: Front Psychiatry. 2022 Oct 14;13:992657. doi: 10.3389/fpsyt.2022.992657 (PMC9613938; doi:10.3389/fpsyt.2022.992657)
Supplement: Supplementary file 1 [file Data_Sheet_1.docx]

Participants had to react to monetary reward cues with the push of a button. Cues for monetary gain were pictures of 1-Euro coins on a green background. Cues for monetary loss were pictures of 1-Euro coins on a red background. First, a monetary reward cue or a neutral cue (black background) was presented. In order to gain or avoid losing the money, the participants had to press the button. The button had to be pressed within the time the target cue (white square) was displayed. The participants were instructed to press the button accordingly in trials with neutral cues. The period between monetary reward or neutral cues and the target cue was delayed by the presentation of a white cross. The delay is required to elicit and record the anticipation of gain or loss. After target presentation, a feedback indicated the outcome of the present trial and the total outcome for all trials. One run consisted of 27 gain, 27 loss and 18 neutral cues. The cues were randomly ordered for each run. The participants were told that the practice run is only for practicing purpose, that it is easier than the test runs and that the outcome of the practice run is not representative for the task.

Variables of subjective reward processing and Reward Dependence for individuals who smoke (n=13) and are cannabis dependent (n=13).

|  | **Nicotine** | | **Cannabis** | |
| --- | --- | --- | --- | --- |
|  | M (SD) |  | M (SD) |  |
| Motivation | 8.1 (1.3) |  | 7.7 (1.1) |  |
| Gain expectancy | 5.4 (2.4) |  | 4.6 (1.3) |  |
| Effort gain | 6.9 (1.6) |  | 6.1 (1.4) |  |
| Fear loss | 6.5 (2.6) |  | 6.4 (1.8) |  |
| Satisfaction gain | 5.0 (2.6) |  | 6.1 (2.7) |  |
| Reward Dependence (TCI) | 15.2 (3.6) |  | 16.0 (3.5) |  |

Correlations subjective reward processing, nicotine and cannabis use and brain activity.

| **Correlation** | **Study group** | r | *p^*^* |
| --- | --- | --- | --- |
| Cigarettes per day – Motivation | smokers | -0.67 | 0.012 |
| Feedback loss IFG – Cigarettes per day |  | -0.58 | 0.036 |
| Feedback loss IFG – Motivation |  | 0.75 | 0.003 |
| Feedback gain Insula – Gain expectancy | smokers and cannabis dependents | -0.55 | 0.004 |
| Feedback gain Insula – Satisfaction |  | 0.47 | 0.014 |
| Anticipation g vs. l ACC – Gain expectancy | cannabis dependents | -0.66 | 0.015 |
| Gain expectancy – Satisfaction |  | -0.73 | 0.005 |
| Gain expectancy – Abstinence |  | -0.57 | 0.041 |
| Satisfaction – Gram per week |  | -0.59 | 0.035 |
